# CT引导下医用胶在胸腔镜术前单侧多个或单一肺结节定位的应用

**DOI:** 10.3779/j.issn.1009-3419.2021.102.52

**Published:** 2022-01-20

**Authors:** 晓刚 谭, 宝东 刘, 毅 张

**Affiliations:** 100053 北京，北京首都医科大学宣武医院胸外科 Department of Thoracic Surgery, Xuanwu Hospital, Capital Medical University, Beijing 100053, China

**Keywords:** 多发肺结节, 医用胶定位, 胸腔镜手术, Multiple pulmonary nodules, Medical glue localization, Thoracoscopy surgery

## Abstract

**背景与目的:**

肺结节定位关系到能否精准、快捷找到并切除病灶，是微创胸腔镜手术（video-assisted thoracic surgery, VATS）成功的重要环节。本研究探讨单一肺结节与两枚以上肺结节VATS术前计算机断层扫描（computed tomography, CT）引导下医用胶定位的可行性，并与单个结节定位的准确性、安全性进行比较。

**方法:**

回顾2018年11月-2021年3月我院VATS术前行CT引导下医用胶定位单侧肺部结节患者的临床资料，按定位结节的数量分为单一结节定位组与多个结节定位组（定位结节数≥2个）。对比两组患者定位时间、成功率、并发症发生率等。

**结果:**

两组共126例结节，其中单一结节定位组62例，多个结节定位组64例。平均单个结节定位时间单一结节定位组为（13.23±4.5）min，多个结节定位组为（10.52±2.8）min，两组间差异有统计学意义（*P* < 0.05）。单一结节定位组定位成功率为100%，多个结节定位组为98.4%，两组无统计学差异（*P* > 0.05）。定位后所有胸腔镜手术均顺利完成。多个结节定位组气胸的发生率明显高于单一结节定位组（*P*=0.07）。

**结论:**

与单侧肺结节定位相比，单侧CT引导下胸腔镜下医用胶定位多发性肺结节也是可行和准确的，值得临床应用。但应注意气胸发生率较高。

## Abstract (en)

**Background and objective:**

The localization of pulmonary nodules is related to whether the lesions can be found and removed accurately and quickly. It is an important link for the success of minimally invasive video-assisted thoracic surgery (VATS). This study investigated the feasibility of medical glue localization under VATS video-assisted thoracoscopic computed tomography (CT) guidance for single pulmonary nodule and more than two pulmonary nodules, and compared with the accuracy and safety of single nodule localization.

**Methods:**

A retrospective analysis of the clinical data of patients who underwent unilateral CT-guided medical glue localization before VATS from November 2018 to March 2021 were performed, the patients was divided into multiple pulmonary nodules group (localized nodules ≥2) and single pulmonary nodule group according to the number of localized nodules. The localization time, success rate and complication rate of the two groups were compared.

**Results:**

There were 126 nodules in the two groups, including 62 in single pulmonary nodule group and 64 in multiple pulmonary nodules group. The average single nodule localization time was (13.23±4.5) min in single pulmonary nodule group and (10.52±2.8) min in multiple pulmonary nodules group, the difference between the two groups is statistically significant (*P* < 0.05). The localization success rate of single pulmonary nodule group and multiple pulmonary nodules group were 100% and 98.4% separately, the difference between the two groups was not statistically significant (*P* > 0.05). All VATS were successfully completed after localization. The incidence of pneumothorax was higher in multiple pulmonary nodules group than in single pulmonary nodule group (*P*=0.07).

**Conclusion:**

Compared with localization of single lung nodule, unilateral CT-guided medical glue localization for multiple pulmonary nodules before VATS is also feasible and accuracy, it is worthy of clinical application. But the higher rate of pneumothorax should be paid attention to.

随着社会经济水平不断提髙，人们的健康意识不断增强，低剂量螺旋计算机断层扫描（computed tomography, CT）被用于越来越多的高危人群肺癌筛查，肺小结节发现概率也随之增加^[1]^。肺结节是指直径≤3 cm的肺部病灶，按照其密度均匀与否和实质成分占比，分为纯磨玻璃结节（pure ground-glass nodule, pGGN）、混合磨玻璃结节（mixed ground-glass nodule, mGGN）和实性结节^[2]^。前两种生长相对缓慢，但较肺实性结节恶变概率高^[3-5]^。此外，如pGGN在生长过程中出现实性成分或变为实性结节，此时该结节会有更高的风险转变为浸润性腺癌^[6, 7]^。胸腔镜微创技术（video-assisted thoracic surgery, VATS）患者损伤小，恢复快，对早期肺癌尤其是磨玻璃结节（ground-glass nodule, GGN）具有显著优势，被广泛应用于临床中^[8]^。但是对于直径小于1 cm、距离脏层胸膜超过5 mm的小结节，无法通过术者直视下或手指的触觉以及借助腔镜器械滑行感知进行定位，延长手术探查时间，特别是对于质地柔软的pGGN，更加大手术难度，增加了开胸的风险^[9, 10]^。因而肺小结节定位，关系到能否精准、快捷找到并切除病灶，是VATS肺结节手术治疗成功的重要环节^[11]^。

目前，对胸腔镜术前同期定位肺内多个结节的研究较少。我院从2018年利用医用胶VATS术前定位。医用胶主要成分为氰基丙烯酸酯，遇到水或血液等弱基立即聚合，主要由于对器官、组织创面渗血的封闭、止血。由于此特性医用胶可以在体内迅速固化，确保定位的准确性，同时可阻断血管断端，使血液凝固，从而减少因穿刺导致的漏气和出血，尤其适用于同侧多发结节的定位及手术。定位时，定位针头应放置在肺结节周边10 mm的范围内，如过近，胶弥散可能污染结节，干扰病理诊断；如距离结节较远，切割目标肺组织时可能出现病灶遗漏。本研究旨在评估胸腔镜术前采取CT引导下医用胶定位单侧多个肺结节的可行性，同时与定位单一肺结节的安全性和准确性进行比较。

## 1 资料和方法

### 1.1 临床资料

回顾分析2018年11月-2021年3月我院行胸腔镜术前单侧定位的患者共90例，其中单独一处定位者62例，其中男性22例，女性40例。同期多个定位（≥2个）者28例，男性7例，女性21例。按定位结节的数量分为单一结节定位组与多个结节定位组（定位结节数≥2个）。单一结节定位组年龄31岁-76岁，中位年龄62岁。多个结节定位组年龄39岁-77岁，中位年龄64岁。两组患者平均年龄相仿，差异无统计学意义（*P* > 0.05），多个结节定位组患者中女性比例更高。所有患者共定位结节126个，其中单一结节定位组62个，多个结节定位组64个。患者一般临床资料及结节位置分布情况详见表 1。全部患者心肺功能可耐受VATS肺叶切除手术，无其他手术禁忌，所有入选者均签署知情同意书。

**表 1:** 两组患者的临床资料 Patient demographic information of the two groups patients variable data

Variable	Single pulmonary nodule group (*n*=62)	Multiple pulmonary nodules group (*n*=28)	*t*/*χ*^2^	*P*
Age (yr)	58.5±9.8	55.2±10.7	1.44	0.15
Gender				
Male	20 (32.26%)	7 (25.00%)	0.48	0.49
Female	42 (68.74%)	21 (75.00%)	0.16	0.16
No.of nodules				
1	62	0		
2	0	21		
3	0	6		
4	0	1		
Nodules of lobar location			5.17	0.27
RUL	22 (35.48%)	22 (34.38%)		
RML	4 (6.45%)	8 (12.50%)		
RLL	15 (24.19%)	10 (15.63%)		
LUL	17 (27.42%)	14 (21.88%)		
LLL	4 (6.45%)	10 (15.63%)		
Hospital day (d)	9.0±2.9	9.7±3.4	-1.00	0.32
Localization cost (¥)	2, 200.0±432.5	2, 674.5±481.3	-4.65	< 0.01
SD: standard deviation; No.: number; RUL: right upper lobe; RML: right middle lobe; RLL: right lower lobe; LUL: left upper lobe; LLL: left lower lobe.				

### 1.2 定位穿刺

以多发结节穿刺为例，患者于手术前一天下午或当天术前2 h-3 h定位。根据术前预判定位结节的恶性程度、手术方式、结节大小、数量方面规划穿刺体位以及拟穿刺结节的顺序。尽量相同扫描野，相同体位定位多个结节，如不合适，首先穿刺预计恶性程度最高的结节。依据“垂直就近”^[12]^的原则确定进针部位，尽量避开叶裂。常规消毒铺单，2%利多卡因10 mL局部浸润麻醉，采用Siemens 64排螺旋CT对肿瘤进行1 mm薄层扫描以确定穿刺点及穿刺方向，保留麻醉注射器于胸壁上再次扫描。根据注射器进针角度和深度再次校正穿刺点及穿刺方向，用一次性神经阻滞穿刺针（上海埃斯埃医械塑料制品有限公司）连接1 mL注射器进行穿刺。注射器内预先抽取0.2 mL-0.3 mL福爱乐医用胶（北京福爱乐科技发展有限公司生产），注意排干空气以防空气栓塞，间断进针并重复局部CT扫描直到针尖位置达到脏层胸膜下结节旁1 cm左右，注意不要穿刺到结节, 同法定位其余结节。如CT扫描发现进针点、进针角度存在偏差（肿瘤旁大于2 cm），则予以调整。如定位结节不在同一扫描野内，则更换体位，同法按恶性程度依次行其余结节穿刺定位。根据穿刺角度及深度依次穿入所有的定位针至所规划位置。重复CT扫描，当穿刺针的位置和角度满意，按术前预判结节的恶性程度高低依次缓慢注射医用胶水0.2 mL-0.3 mL。若肿瘤位置较深注射医用胶水可适量增多，可以边注射边退针至肺外。注射后再次复查全胸部CT，此时可了解是否气胸、肺内出血以及定位胶与肿瘤的相对位置关系。如在CT肺窗上见到新出现的高密度阴影，定位效果满意，无明显气胸及出血，将患者送至手术室或者病房等待手术。

### 1.3 VATS手术

单孔胸腔镜腋前线第4肋间3 cm-4 cm切口，双孔胸腔镜腋前线第3或第4肋间3 cm切口为操作孔，腋中线第7肋间为观察孔。胸腔镜下全面探查胸腔，通过操作口进行触诊，结合定位影像确定结节的位置，并以电凝钩烧灼或系线标记病灶，如胶邻近脏层胸膜，亦可看到表面胸膜皱缩或残留凝固胶水。根据定位胶与病灶的解剖位置关系，先以卵圆钳夹持定位胶包含结节以及周围少量正常肺组织，用内镜直切割缝合器楔形切除病灶送术中快速病理。如结节位置较深，根据术前三维重建行相应部位肺段切除术。按定位胶指示位置，切除标本剖开后可以方便找到病灶，如冰冻病理为浸润性肺腺癌，按照患者心肺功能及同期切除肺结节数量，对侧肺内有无结节等评估肺功能损失情况，继续行肺叶切除术或只行肺楔形切除术。

### 1.4 统计方法

组间比较采用*t*检验，计量资料以均数±标准差（Mean±SD）表示，计数资料组间比较采用*χ*^2^检验或*Fisher*精确检验。采用SPSS 20.0软件对数据进行统计学分析。*P* < 0.05为差异有统计学意义。

## 2 结果

### 2.1 两组患者定位结果

两组患者共定位肺部结节126个，其中多个结节定位组中1例患者术中右下肺未发现定位胶，根据解剖定位楔形切除，肺标本上找到结节，判定为定位失败。单一结节定位组定位成功率100%，多个结节定位组98.4%，两组间定位成功率的差异无统计学意义（*P* > 0.05），定位后所有胸腔镜手术均顺利完成。平均单个结节定位时间单一结节定位组为（13.23±4.50）min，多个结节定位组为（10.52±2.80）min，定位单个结节平均耗时多个结节定位组较单一结节定位组更短，且此差异有统计学意义（*P* < 0.05）。

### 2.2 两组患者手术及术后病理结果

单一结节定位组患者行单纯楔形切除共43例，单纯段切10例，单纯叶切9例，多个结节定位组患者段切+楔形切除6例，叶切+段切2例，楔形+楔形切除20例。单一结节定位组结节平均直径较多个结节定位组大，差异均有统计学意义（*P* < 0.05）。所有切除的结节中，单一结节定位组浸润性腺癌15个，腺癌伴微浸润5个，原位腺癌24个，不典型腺瘤样增生4个，甲状腺癌转移瘤1例，乳腺癌转移瘤1例，其余良性（错构瘤、硬化性肺细胞瘤、淋巴组织增生、肉芽肿性炎等）共12个。多个结节定位组浸润性腺癌7个，腺癌伴微浸润4个，原位腺癌26个，不典型腺瘤样增生7个，其余良性共20个，详见表 2。均接受电视胸腔镜手术，无因结节探查困难而中转开胸，无并发症发生。恶性的比例单一结节定位组为80.6%（50/62），多个结节定位组为68.75%（44/64）。

**表 2:** 两组术式、术后病理及并发症比较 Comparasion of operation, pathology and complication

Variable	Single pulmonary nodule group (*n*=62)	Multiple pulmonary nodules group (*n*=64)	*t*/*χ*^2^	*P*
Localization success rate	62 (100.00%)	63 (98.40%)	0.98	0.32
Localization time (min)	13.23±4.50	10.52±2.80	3.48	< 0.01
Tumor size (cm)	0.91±0.36	0.75±0.31	2.68	0.01
Pleural distance (cm)	1.34±0.81	1.58±0.70	-1.78	0.08
Operation				
Wedge resection	43 (69.35%)	0		
Segmentectomy	10 (16.13%)	0		
Lobectomy	9 (14.52%)	0		
Segmentectomy+wedge resection	0	6 (21.43%)		
Lobectomy+wedge resection	0	2 (7.14%)		
Wedge resection+wedge resection	0	20 (71.43%)		
Pathology			5.08	0.02
Benign	12 (19.35%)	24 (37.50%)		
Malignant tumor	50 (80.65%)	40 (62.50%)		
Complication				
Irritable cough	16 (25.81%)	6 (21.43%)	0.20	0.66
Pneumothorax	8 (12.90%)	8 (28.57%)	3.24	0.07

### 2.3 两组患者定位并发症及处理

穿刺定位时造成的并发症主要为刺激性咳嗽和气胸。气胸发生率单一结节定位组为12.7%（8/62），多个结节定位组为29.6%（8/28），多个结节定位组气胸的发生率明显高于单一结节定位组，两组无统计学差异（*P*=0.07）。均无症状且无需穿刺或闭式引流处理。刺激性咳嗽发生率单一结节定位组为25.8%（16/62），多个结节定位组为21.4%（6/28），口服止咳药物缓解。两组均无血胸和空气栓塞等严重并发症。

## 3 讨论

近年来临床上多发肺结节的诊断比例逐渐增高，尤其是影像学表现为磨玻璃样病变的多发结节更为常见。目前国际上针对多原发结节的诊治主要参考2013年美国胸科医师协会（American College of Chest Physicians, ACCP）第三版的肺癌诊治^[13]^指引，国内外均形成了选择性积极干预的共识。然而多发肺结节在手术干预的过程中有其特殊性，比较突出的即为本研究所涉及的术前定位问题^[14-16]^。

术前CT引导定位有微弹簧圈定位、Hook-wire定位、亚甲蓝定位及医用胶定位等。微弹簧圈定位法由于弹簧圈体积小，触摸感觉具有一定困难，为确保手术切除的准确性，手术过程中需要X线透视，这在一定程度上会增加手术辐射量及难度。亚甲蓝易弥散，这就要求胸腔镜手术最佳时间是在定位注射后的3 h内，这给手术安排和衔接带来了不便。而且由于其容易扩散，手术间隔时间较长使得肺表面定位区域变大，造成正常肺组织不必要的损失。医用胶形成的颗粒体，与亚甲蓝等注射液体相比，在体内保留时间更长，使手术衔接更为从容。Huang等^[17]^对113例患者实行了CT引导下医用胶定位和胸腔镜下结节切除手术，成功率为100%，术后有8%的患者出现轻微气胸，另有少数患者出现了疼痛和咳嗽，但均自行好转，此研究证明了使用医用胶进行术前定位的有效性和安全性。

本研究中，无论是单一结节定位组还是多个结节定位组，女性患者例数均高于男性，尤其在多个定位组中，女性例数更是达到了男性的3倍（21/7），这与既往研究中女性肺部多发结节患者更多的情况^[18]^相符。两组患者共定位肺部结节126个，其中多个结节定位组中1例患者术中右下肺未发现定位胶，根据解剖定位楔形切除，肺标本上找到结节，判定为定位失败。因此次定位较早，属于医用胶定位学习曲线阶段，只注射了0.1 mL，量较少，推测胶在肺内弥散以致触摸不着，后来我们常规注射医用胶水0.2 mL-0.3 mL，再无出现此种情况。两组中，结节定位的成功率差异均无统计学意义，表明了采用该方法进行同期多个结节的穿刺定位的成功率与定位单个结节无明显差异。

如同一体位定位多个结节，先定位依次置入定位针，最后注入胶水，而不是逐一重复操作，这种方法能减少患者在CT引导定位时所受的辐射及定位所用时间，结果中多个定位组平均每个结节定位所用的时间小于单个定位组相应的时间也验证了这一点。穿刺时我们尽量调整患者的位置，即患者与扫描床呈一定角度，减少定位针所需穿过的肺组织，尽量垂直穿刺点皮肤入针，降低操作难度（图 1A）。如病灶距离胸膜太深，或最佳穿刺路径被肩胛骨或肋骨遮挡，尽量缩小最终定位针延长线与病灶的垂直距离，必要时可跨肺叶穿刺进针（图 1B）。由于切割闭合器为直线不能弯曲，故定位距脏层胸膜较表浅结节时，胶定位在楔形切除的结节的前方和深面，这样如果切除了定位胶，病灶结节也就肯定切除了。多发结节的定位很难保证100%成功，为避免气胸造成后续无法顺利定位，按照手术的目的，建议术前影像学恶性程度高的结节优先定位。

**图 1:**
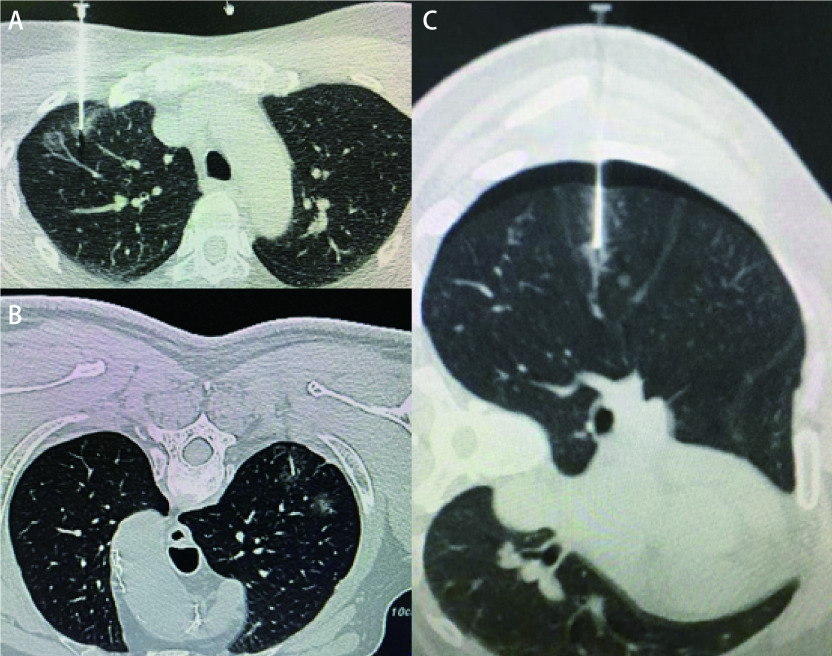
CT引导下医用胶在胸腔镜术前肺结节定位。A：依据“垂直就近”的原则定位；B：最佳穿刺路径被肩胛骨或肋骨遮挡，医用胶也可准确定位；C：发生气胸时，定位中叶结节。 The application of CT-guided localization with medical glue for small pulmonary nodules before VATS. A: Localization according to the principle of "vertical proximity"; B: The medical glue can be accurately localizated, although the best puncture path is blocked by shoulder blades or ribs. C: When pneumothorax occurs, locate the middle lobe nodule. CT: computed tomography; VATS: video-assisted thoracic surgery.

术前患者定位时并发症主要表现为刺激性咳嗽以及少量的气胸，均无需紧急手术或立即胸腔闭式引流，但多个定位组的气胸发生率明显高于单个定位组，这需要引起注意。定位多个结节时，尽量同一体位置入定位针，先不刺入胸膜，在胸壁调整到最佳角度，再根据深度穿入定位针至规划位置。最后注入胶水，凝固的胶水也同时封闭漏气的针眼，而不是逐一重复操作。故多发结节定位气胸大部分发生在拔出穿刺针之后，可能第一针也可能后续几针拔出才出现气胸，但已经不影响穿刺的成功。但也有少数情况，定位多发结节时，穿刺第一针或第二针就出现气胸，由于定位针较细（22 G），一般肺压缩程度并不严重，此时预判恶性程度最高的结节定位针应该置入理想位置。如定位右侧肺，我们会先定位右上或右下肺结节，最后定位中叶结节，因为如发生气胸右上或右下肺压缩程度较中叶小。气胸时，肺的弹性下降，向胸膜进针时没有突破感；而且由于肺的萎陷，定位针进入肺的深度会比我们预测的深度浅。这时在重新CT扫描后，计算胸壁到肺门的安全距离，保证安全的前提下用力进针，此时大概率针尖的深度较结节深，根据CT扫描的距离相应拔出至针尖到结节大致理想距离（图 1C）。虽然定位操作中会尽量减少定位针进出胸膜的次数，但是定位多发结节的气胸发生率随着定位次数的增加而明显增加，当定位结节大于5个时气胸发生率达到了100%^[19]^。为避免穿刺次数过多导致气胸发生率提高，我们建议同期不进行3枚以上结节的定位。

本研究切除的所有结节中单一结节定位组结节平均直径较多个结节定位组大，病理性质恶性的比例也高，差异均有统计学意义。单一结节定位组为80.6%（50/62），多个结节定位组为62.5%（40/64）。多发结节在被切除的良性结节中，一部分是因同侧的主病灶为恶性需手术，同时患者要求将所有可能潜在风险的结节均切除，因此术前对结节良恶性判断的准确程度不能仅以此数据下结论。患者对于肺部结节过度焦虑的情况较为常见，外科医师应尽量避免进行不必要的手术干预。由于单一结节定位组病理性质恶性的比例高，肺叶及肺段切除的手术也相应较多，故单一结节定位组平均住院日并未较多个结节定位组明显减少，但术前定位费用多个结节定位组较单一结节定位组高。

综上，肺部小结节术前辅助定位技术大大提高了VAST肺结节切除术的成功率，降低了微创术中转开胸的机率，具有重要的临床意义。VATS术前CT引导下医用胶定位多个肺部结节的方法安全可行，且与单个结节定位相比准确性无明显差异，但气胸发生率较高，定位时需引起重视。
